# Methotrexate-conjugated zinc oxide nanoparticles exert a substantially improved cytotoxic effect on lung cancer cells by inducing apoptosis

**DOI:** 10.3389/fphar.2023.1194578

**Published:** 2023-10-17

**Authors:** Prakriti Mishra, Mohd Faizan Ali Ahmad, Lamya Ahmed Al-Keridis, Mohd Saeed, Nawaf Alshammari, Nadiyah M. Alabdallah, Rohit Kumar Tiwari, Afza Ahmad, Mahima Verma, Shireen Fatima, Irfan Ahmad Ansari

**Affiliations:** ^1^ Department of Biosciences Integral University Lucknow, Lucknow, India; ^2^ Biology Department, Faculty of Science, Princess Nourah Bint Abdulrahman University, Riyadh, Saudi Arabia; ^3^ Department of Biology, College of Science, University of Hail, Hail, Saudi Arabia; ^4^ Department of Biology, College of Science, Imam Abdulrahman Bin Faisal University, Dammam, Saudi Arabia; ^5^ Basic and Applied Scientific Research Centre, Imam Abdulrahman Bin Faisal University, Dammam, Saudi Arabia; ^6^ Department of Clinical Research, School of Allied Health Sciences, Sharda University, Uttar Pradesh, India

**Keywords:** zinc oxide, nanoparticles, methotrexate, lung cancer, A549, reactive oxygen species, caspases

## Abstract

In the current study, we report the synthesis of methotrexate-conjugated zinc oxide nanoparticles (MTX-ZnONPs) and their high efficacy against lung cancer cells. Conjugation of MTX with ZnONPs was authenticated by UV-vis spectroscopy, dynamic light scattering (DLS), Fourier-transform infrared (FTIR) spectroscopy, and transmission electron microscopy (TEM). This drug-nanoconjugate also showed high drug-loading efficiency. The therapeutic efficacy of MTX-ZnONPs was further tested *in vitro* against A549 cells, and the results of MTT and LDH release assays showed that MTX-ZnONPs, in addition to free MTX, were efficient in exerting cytotoxic effect on A549 cells; however, the effectiveness of MTX-ZnONPs was found to be considerably enhanced at very low doses compared to that of free MTX. Moreover, ZnONPs alone significantly inhibited the cell viability of A549 cells at a much higher concentration compared to MTX-ZnONPs and MTX. Furthermore, the cytomorphology of A549 cells was characterized by cellular shrinkage and detachment from the surface in all the treatment groups. Similarly, A549 cells, in all the treatment groups, showed fragmented and condensed nuclei, indicating the initiation of apoptosis. Mitochondrial membrane potential (ψ_m_) in A549 cells showed a gradual loss in all the treatment groups. Results of the qualitative and quantitative analyses depicted increased reactive oxygen species (ROS) levels in A549 cells. The results of the caspase activity assay showed that MTX-ZnONPs andfree MTX caused significant activation of caspase-9, -8, and -3 in A549 cells; however, the effect of MTX-ZnONPs was more profound at very low doses compared to that of free MTX. Thus, our results showed high efficacy of MTX-ZnONPs, suggesting efficient intracellular delivery of the drug by ZnONPs as nanocarriers.

## Introduction

Despite recent advancements in molecularly targeted therapy that have fundamentally transformed the therapeutic strategies for lung cancer, chemotherapy is still a key component of lung cancer treatment (Lee, 2019). Whether administered alone or in conjunction with other treatment options, chemotherapy has benefitted patients with early- and late-stage lung cancer in terms of survival and quality of life. Combination therapy with other classes of anticancer agents and new drug delivery systems haves shown promising results, and these are currently the focus of ongoing research. The ultimate objective of researchers is to increase therapeutic efficacy and reduce the potential risks associated with a drug.

Methotrexate (MTX) is a well-established drug, widely used in numerous cancer treatments, psoriasis, and rheumatological diseases (Khan et al., 2012). Currently, many forms of cancer, such as osteosarcoma, acute lymphoblastic leukemia, acute myeloid leukemia, meningeal leukemia and lymphoma, non-lymphoma, Hodgkin’s lymphoma, breast cancer, and bladder cancer, are treated with MTX in conjunction with other drugs (Frei et al., 1975; Evans et al., 1986; Huennekens, 1994; Crews et al., 2004). MTX is a folic acid analog that inhibits dihydrofolate reductase activity, thereby disrupting cellular folate metabolism (Aghajanzadeh et al., 2018). MTX has been shown to exhibit therapeutic efficacy against numerous types of cancer cells that have overexpress folate receptors on their surface (Duthie, 2001).

The bioavailability of chemotherapeutic drugs, which can cause severe toxicities and off-target side effects, is one of the major challenges in cancer chemotherapy (Gewirtz et al., 2010). One plausible approach to overcome these challenges is the design of a nanocarrier that can selectively deliver cytotoxic doses of therapeutic drugs to cancer cells (Nosrati et al., 2017; Aghajanzadeh et al., 2018; Zamani et al., 2018). Zinc oxide nanoparticles (ZnONPs), one of the nanocarriers used for drug delivery and cancer therapy, have attracted significant interest in drug delivery systems (Barui et al., 2018). ZnONPs are considered as potential candidates for drug delivery due to their easy synthesis from low-cost precursors, biocompatibility, and effective cellular internalization through endocytosis (Duncan and Richardson, 2012; Singh et al., 2020).

When compared to conventional methods, formulation and targeting techniques for MTX using controlled release carriers, multi-articulate approaches, prodrugs, and drug conjugates, have been found to increase bioavailability, minimize adverse effects, and maximize clinical effectiveness (Khan et al., 2012). Previous studies have reported high expression of folate receptors on lung cancer cells (Nunez et al., 2012; O’Shannessy et al., 2012). Moreover, MTX has some affinity for folate receptors because of its structural similarity with folic acid (Xie et al., 2018). Moreover, there is no report to date showing the conjugation of MTX with ZnONPs. Therefore, we hypothesized that due to the high expression of folate receptors on lung cancer cells, MTX-conjugated nanoparticles could potentially be efficiently internalized inside cancer cells and show an increased cytotoxic effect against lung cancer cells. Accordingly, to test this hypothesis, we synthesized and investigated the efficacy of MTX-conjugated ZnO nanoparticles (MTX-ZnONPs) against an A549 non-small cell lung adenocarcinoma cell line.

## Materials and methods

### Materials

Methotrexate, 1-ethyl-3-(3-dimethyl) carbodiimide (EDC), 2′,7′-dichlorodihydrofluorescein diacetate (H_2_DCFDA), 4′,6-diamidino-2-phenylindole (DAPI), 3-(4,5-dimethylthiazol-2-yl)-2,5-diphenyltetrazolium bromide (MTT), and Rhodamine 123 were purchased from Sigma-Aldrich (St. Louis, United States). Dulbecco’s Modified Eagle Medium, antibiotic–antimycotic solution (penicillin, streptomycin, and amphotericin B), fetal bovine serum (FBS), and LDH Cytotoxicity Assay Kit were procured from Gibco (Thermo Fisher Scientific, Waltham, United States). Caspase-9, -8, and -3 assay kits were procured from BioVision(Milpitas, United States). All other chemicals were of analytical grade.

### Methods

#### Biosynthesis and characterization of ZnONPs

The synthesis process of the fungus (*Aspergillus niger OL*636020)-mediated ZnONPs and their characterization by FTIR, DLS, zeta potential, TEM, and UV-vis spectroscopy analysis were discussed in our previous publication [13].

#### Conjugation of MTX with ZnONPs

MTX was conjugated with biogenically synthesized ZnONPs. The conjugation was achieved using an EDC coupler, which reacts with carboxyl groups present on ZnONPs and the amino group of MTX to produce MTX-ZnONPs. Briefly, 1 mL of the reaction mixture was prepared by adding 50 mM HEPES buffer, MTX (3 mg/mL), 800 µL of ZnONP suspension, and 5 mM EDC. The mixture was incubated for 5 h at 30°C. Finally, MTX-ZnONPs were separated via centrifugation at 10,000 rpm for 10 min.

#### Drug loading efficiency of ZnONPs

The loading percentage of MTX on ZnONPs was determined by measuring the absorbance before and after conjugation at 302 nm. The loading efficiency of the nanoparticle was calculated using the following formula:

$$Percent\;Loading\;of\;MTX\;on\;ZnONPs=\left[\left(A-B\right)\right]\times \frac{100}{A},$$

where A represents the absorbance of the whole drug added to ZnONPs and B represents the absorbance of the unbound drug in the supernatant of the drug–nanoparticle conjugate solution.

### Characterization of MTX-conjugated ZnONPs

MTX-ZnONPs were characterized by UV-vis spectrophotometry (Shimadzu dual-beam spectrophotometer, model UV-1601 PC, 1 nm resolution. Kyoto, Japan). The mean particle size and zeta potential of MTX-ZnONPs were determined using a particle size analyzer (Zetasizer Nano-ZS, Model ZEN3600, Malvern Instruments Ltd., Malvern, United Kingdom) [(Gomathy and Sabarinathan, 2010), 15]. Transmission electron microscope (Tecnai™ G2 Spirit Bio-TWIN, FEI, Hillsboro, United States) was used to investigate the size of the inorganic core at a accelerating voltage of 80 kV (Khan S. et al., 2018). The binding confirmation of MTX on the surface of ZnONPs was analyzed by Fourier-transform infrared spectroscopy (FTIR) (Shimadzu FTIR-8201 PC infrared spectrophotometer) operating in the diffuse reflectance mode at a resolution of 4 cm^−1^. A total of 256 scans of the ZnONPs film (400–4,000 cm^−1^ range) were acquired to obtain good signal-to-noise ratios.

### Cell culture

A549 cells were purchased from the National Centre for Cell Science (NCCS), Pune, India. The cells were cultured in DMEM, supplemented with 10% FBS and 1% antibiotic–antimycotic solution, in a humidified atmosphere containing 5% CO_2_ at 37°C.

### Assessment of cytotoxicity

To assess the cytotoxic effect of MTX-ZnONPs andMTX and ZnONPs alone, A549 cells (5 × 10^3^ per well) were treated with various doses of MTX-ZnONPs and MTX and ZnONPs alone, for 24 h. Then, 10 μL of MTT dye (5 mg/mL in PBS) was added to each well, followed by incubation for 2 h. Later, 100 μL DMSO was added to each well to solubilize the formazan crystals formed by viable cells. Finally, the absorbance of each well was measured at 570 nm using a microplate reader (Bio-Rad, United States). The results were expressed as percent cell viability, which was calculated using the following formula:

$$Cell\;viability\;\left(\%\right)=\frac{Average\;of\;At}{Average\;of\;Ac}\times 100,$$

where *At* and *Ac* are the absorbances of the treated and untreated control wells, respectively.

### LDH cytotoxicity assay

Briefly, A549 cells (5 × 10^3^ per well) were placed in a 96-well plate, then co-cultured with various doses of MTX-ZnONPs and MTX and ZnONPs alone for 24 h. Subsequently, LDH activity was measured in all the treatment groups, as per the manufacturer’s instructions. Then, the percent cytotoxicity in A549 cells in all the treatment groups, was calculated using the following formula:

$$\%\;Cytotoxicity=\frac{\left(Drug\;Treated\;LDH\;activity\right)-\left(Spontaneous\;LDH\;activity\right)}{Maximum\;LDH\;activity-Spontaneous\;LDH\;activity}\times 100.$$

### Examination of the morphological alterations

Briefly, A549 cells (5 × 10^3^ per well) were co-cultured with isoeffective doses (IC_25_, IC_50_, and IC_75_) of MTX-ZnONPs, MTX alone, and ZnONPs alone for 24 h. Subsequently, the changes in cellular morphology of cells in all the treatment groups were examined, and images were taken using the relief phase channel of the FLoid imaging station (ThermoScientific, United States).

### Assessment of nuclear changes

Nuclear changes in A549 cells were assessed by staining with DAPI dye. The cells (1 × 10^4^ per well) were treated with isoeffective doses of MTX-ZnONPs, MTX alone, and ZnONPs alone for 24 h, followed by washing with PBS and fixing in 4% paraformaldehyde for 10 min. Cells were permeabilized with permeabilizing buffer (0.25% Triton X-100) and, subsequently, stained with DAPI dye (300 nM). Finally, the cells were observed under a blue filter using the FLoid imaging station (ThermoScientific, United States).

### Analysis of mitochondrial membrane potential (ΔΨm)

Mitochondrial membrane potential in lung cancer cells was examined via Rhodamine 123 staining. Briefly, A549 cells (1 × 10^5^ cells per well) were grown in a 24-well plate, followed by treatment with isoeffective concentrations of MTX-ZnONPs and MTX and ZnONPs alone for 24 h. Later, the cells were washed and stained with Rhodamine 123 (300 nM) for 30 min in the dark, and photomicrographs were taken using a FLoid imaging station (ThermoScientific, United States).

### Detection and quantification of cytosolic reactive oxygen species (ROS)

The cytosolic ROS in A549 cells was detected by a fluorogenic dye (H_2_DCFDA). Briefly, A549 cells (5 × 10^4^ per well) were treated with isoeffective doses of MTX-ZnONPs as well as MTX and ZnONPs alone for 6 h. Thereafter, the cells were stained with H_2_DCFDA (25 µM) for 30 min at 37°C, followed by washing of excess dye with phosphate-buffered saline (PBS). Finally, photomicrographs were captured using the green fluorescence channel of a FLoid imaging station (Thermo Scientific, United States).

For the quantification of cytosolic ROS, A549 cells (1 × 10^4^ per well) in a 96-well black-bottomed culture plate were treated with various doses of MTX-ZnONPs, MTX alone, and ZnONPs alone for 6 h, followed by incubation with H_2_DCFDA (25 µM) for 30 min at 37°C. Fluorescence intensity was measured using a multi-well microplate reader (Synergy H1 hybrid multi-mode microplate reader, BioTek, United States). Values were shown as percent DCF fluorescence relative to the untreated control.

To further confirm the role of ROS in drug–nanoparticle conjugate-induced apoptosis, A549 cells were pretreated with 5 mM NAC for 2 h, followed by various doses of MTX-ZnONPs, MTX alone, and ZnONPs alone for another 24 h. Thereafter, cell viability was determined in each treatment group by MTT assay.

### Measurement of caspase activity

The activation of caspases in A549 cells was determined using caspase-9, -8, and -3 assay kits to find out the mode of apoptosis in lung cancer cells. Briefly, A549 cells (1 × 10^6^) were co-cultured with various doses of MTX-ZnONPs, MTX alone, and ZnONPs alone for 24 h. Later, caspase activity was measured as per the manufacturer’s protocol.

### Evaluation of the effect of caspase inhibitors

Caspase-3 inhibitor was used to confirm the activation of caspases and ultimately program cell death in lung cancer cells due to drug–nanoparticle conjugate treatment. Briefly, A549 cells were pretreated with 50 μM of each caspase inhibitor for 2 h, followed by treatment with various doses of MTX-ZnONPs, MTX alone, and ZnONPs alone for another 24 h. Finally, cell survival in each treatment group was evaluated using the MTT assay.

### Statistical analysis

The data are represented as the mean ± SEM of three separate experiments that were carried out in triplicate. One-way ANOVA with Dunnett’s *post hoc* test, two-way ANOVA with Bonferroni’s *post hoc* test, and paired *t*-test (GraphPad Prism 8.0) were used to determine significance in various treatment groups (*p < 0.05, **p < 0.01, and ***p < 0.001 denote significant differences between means of various treatment groups).

## Results

### Characterization of drug–nanoparticle conjugates

**FIGURE 1:**
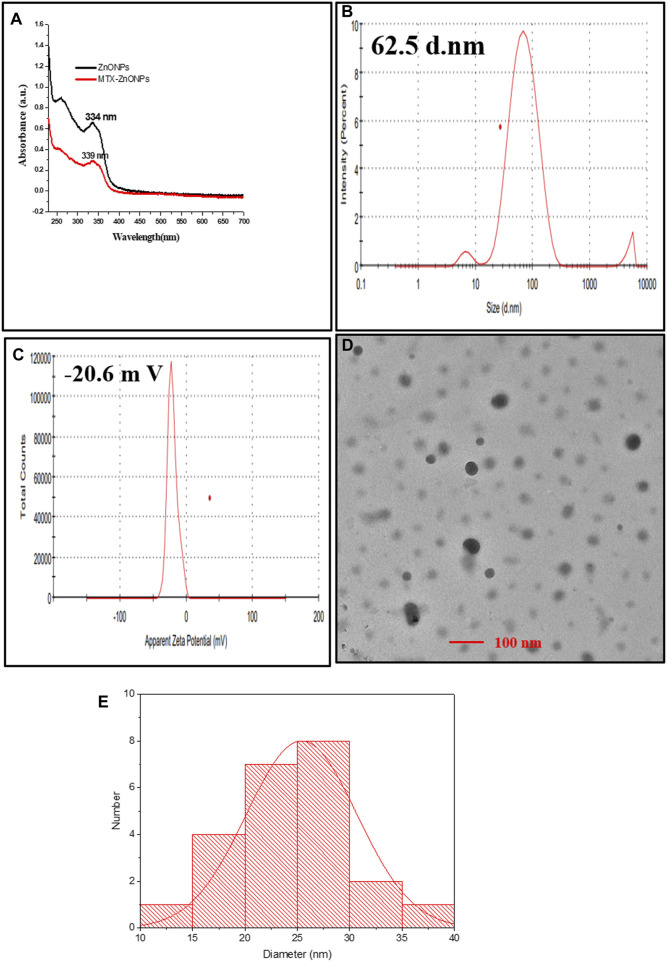
Characterization of MTX-ZnONPs under **(A)** UV-visible spectra (339 nm), **(B)** hydrodynamic diameter (62.5 nm), **(C)** zeta potential (−20.6 mV), and **(D)** transmission electron microscopy (size 25.4 nm). **(E)** Size distribution graph showing the size of MTX-ZnONPs (25.407 ± 5.295 nm).

Conjugation of MTX with ZnONPs was characterized by observing the spectra of drug–nanoparticle conjugate solution, which depicted a shift of the characteristic wavelength of ZnONPs (334 nm to 339 nm) (Figure 1A). Furthermore, the hydrodynamic diameter of drug–nanoparticle conjugates was found to be 62.5 nm, which was higher than that of ZnONPs (53.28 nm) (Figure 1B). The zeta potential of MTX-ZnONPs was observed to be −20.6 mV, slightly higher than that of ZnONPs (−21.4 mV), which describes the good stability of drug-nanoconjugate (Figure 1C). The average size and shape of the MTX-ZnONPs were further validated by TEM. The average size of drug–nanoparticle conjugates, as shown in the size distribution graph, was 25.4 nm, which was greater than that of ZnONPs (17.2 nm) (Figures 1D,E). Moreover, the shape of drug-nanoconjugates was spherical, and they were found to be monodispersed.

**FIGURE 2:**
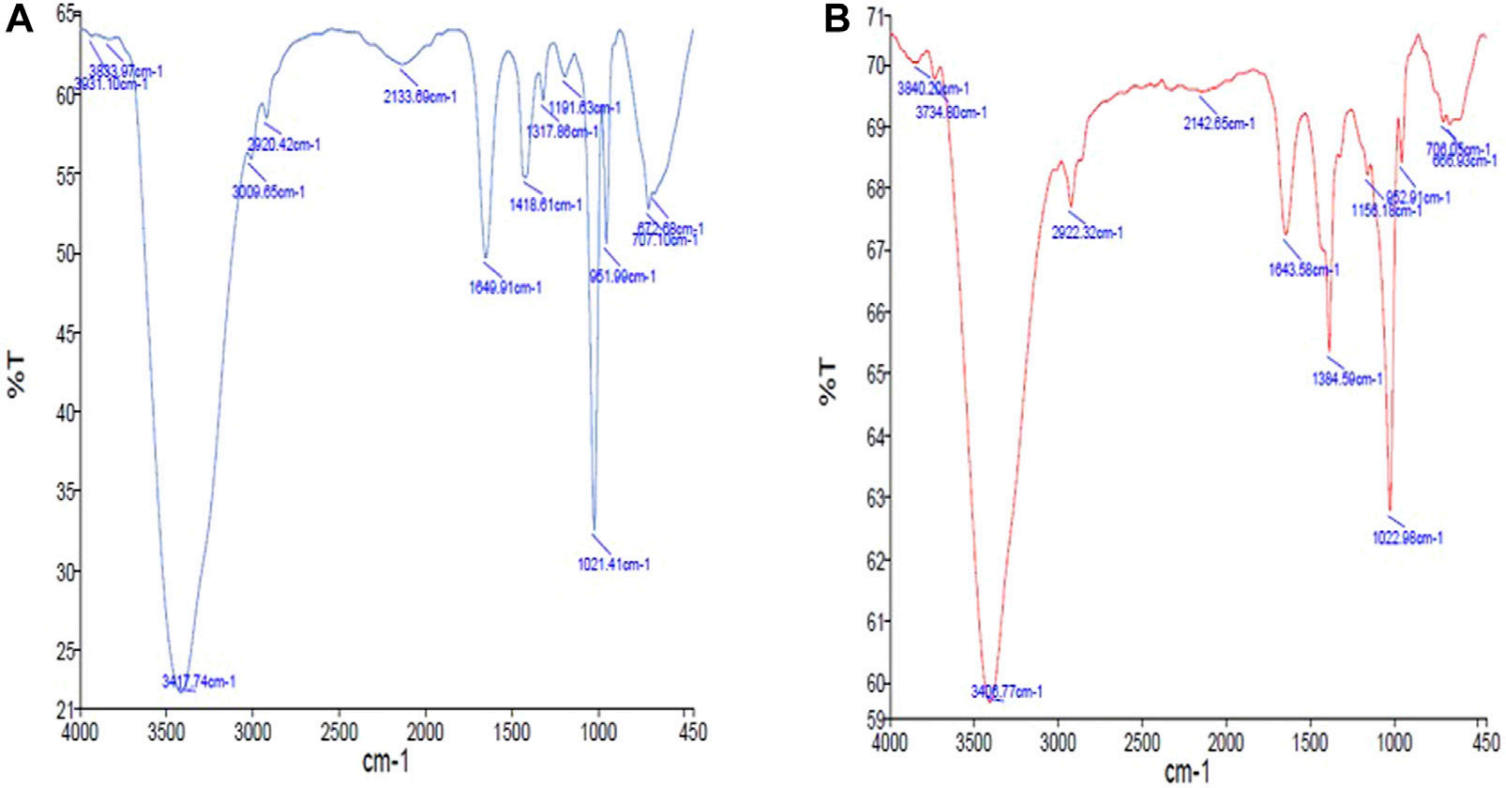
FTIR spectra of **(A)** methotrexate and **(B)** MTX-ZnONPs.

The FTIR spectrum of ZnONPs was shown in our previously published article [13]. In the FTIR spectrum of MTX, the bend peaks of –OH, –NH_2_, and –COOH groups were observed at 3,417 cm^−1^, 1,649 cm^−1^, and 2,133 cm^−1^, respectively (Figure 2B). The stretch peak of the aromatic ring was observed at 2,920 cm^−1^. The FTIR spectrum of the prepared MTX-ZnONPs exhibited strong absorption bands at 3,406 cm^−1^, corresponding to the -OH group stretching (Figure 2C). A new peak was observed at 1,643 cm^−1^, corresponding to the bend peak of the N-H bond. A new peak was also observed at 1,022 cm^−1^, corresponding to the stretchof C-N of the peptide bond, which confirmed the conjugation. Interestingly, in the spectrum of MTX-ZnONPs, the stretch peak of the aromatic ring of MTX was observed at 2,922 cm^−1^. The presence of these peaks in MTX-ZnONPs confirmed the conjugation of MTX with ZnONPs.

### Percent drug loading

The amount of MTX conjugated with ZnONPs was shown as percent drug loading or drug loading efficiency, which was found to be 82.04% ± 3.78%. Thus, the result showed high conjugation of MTX with ZnONPs.

### Cytotoxicity assay

**FIGURE 3:**
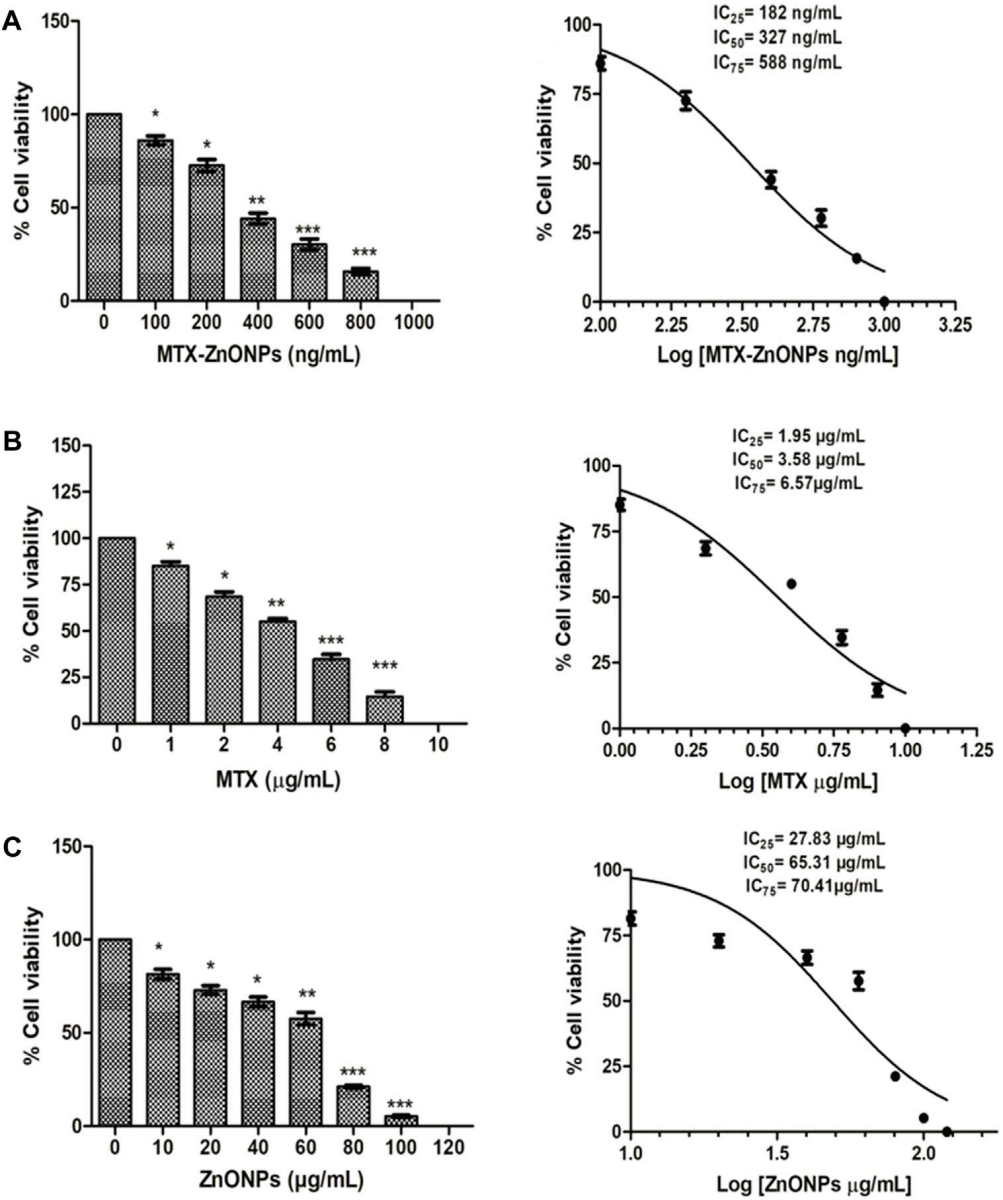
Percent cell viability of A549 cells treated with **(A)** MTX-ZnONPs, **(B)** MTX alone, and **(C)** ZnONPs alone for 24 h assessed by MTT assay. The results presented are the mean ± SEM of three independent experiments performed in triplicate. Significance among different dosage groups were determined using one-way ANOVA followed by Dunnett’s *post hoc* test (where *p < 0.05, **p < 0.01, and ***p < 0.001 represent significant differences compared with vehicle control).

As shown in Figure 4.3, MTX-ZnONPs (100–800 ng/mL) significantly reduced the viability of A549 cells after 24 h of treatment. The cell viability was found to be 86.07% ± 2.37%, 72.6% ± 3.20%, 44.10% ± 2.94%, 30.20% ± 2.93%, and 15.69% ± 1.69% at the doses of 100, 200, 400, 600, and 800 ng/mL MTX-ZnONPs, respectively (Figure 3A). Similarly, MTX alone also significantly inhibited the survival of A549 cells, and the viability was found to be 85.14% ± 2.12%, 68.57% ± 2.54%, 55.03% ± 1.61%, 34.63% ± 2.65%, and 14.59% ± 2.42% at the doses of 1, 2, 4, 6, and 8 μg/mL MTX, respectively (Figure 3B). However, cytotoxic doses of ZnONPs alone for A549 cells were found to be in the range of 10–100 μg/mL, and the cell viability was found to be 81.48% ± 2.53%, 72.97% ± 2.31%, 66.54% ± 2.62%, 57.62% ± 3.32%, 21.18% ± 0.79%, and 5.32 ± 0.55 at the doses of 10, 20, 40, 60, 80, and 100 μg/mL ZnONPs, respectively (Figure 3C). Furthermore, we also performed the cytotoxicity assay of ZnONPs against normal 3T3 cells and found that ZnONPs also significantly inhibited the growth of 3T3 cells at a dose of 10–100 μg/mL (Supplementary Figure S1).

The isoeffective concentrations (IC_25_, IC_50_, and IC_75_) of MTX-ZnONPs were found to be 182 ng/mL, 327 ng/mL, and 588 ng/mL, respectively. Similarly, the IC_25_, IC_50_, and IC_75_ of MTX alone were 1.95 μg/mL, 3.58 μg/mL, and 6.57 μg/mL, respectively. However, the IC_25_, IC_50_, and IC_75_ of ZnONPs alone were 27.83 μg/mL, 65.30 μg/mL, and 70.41 μg/mL, respectively. According to the aforementioned data, the IC_25_ of MTX-ZnONPs was found to be reduced by approximately 10.71-fold, while the IC_50_ was reduced by approximately 10.95-fold compared to MTX alone. Thus, the isoeffective concentrations of MTX-ZnONPs were significantly reduced compared to MTX alone, which substantiated that MTX-ZnONPs exhibited a similar effect on A549 non-small cell lung cancer cells at a much lower concentration compared to MTX. The results also emphasized the efficient delivery of drug-conjugated ZnONPs.

### LDH release assay

**FIGURE 4:**
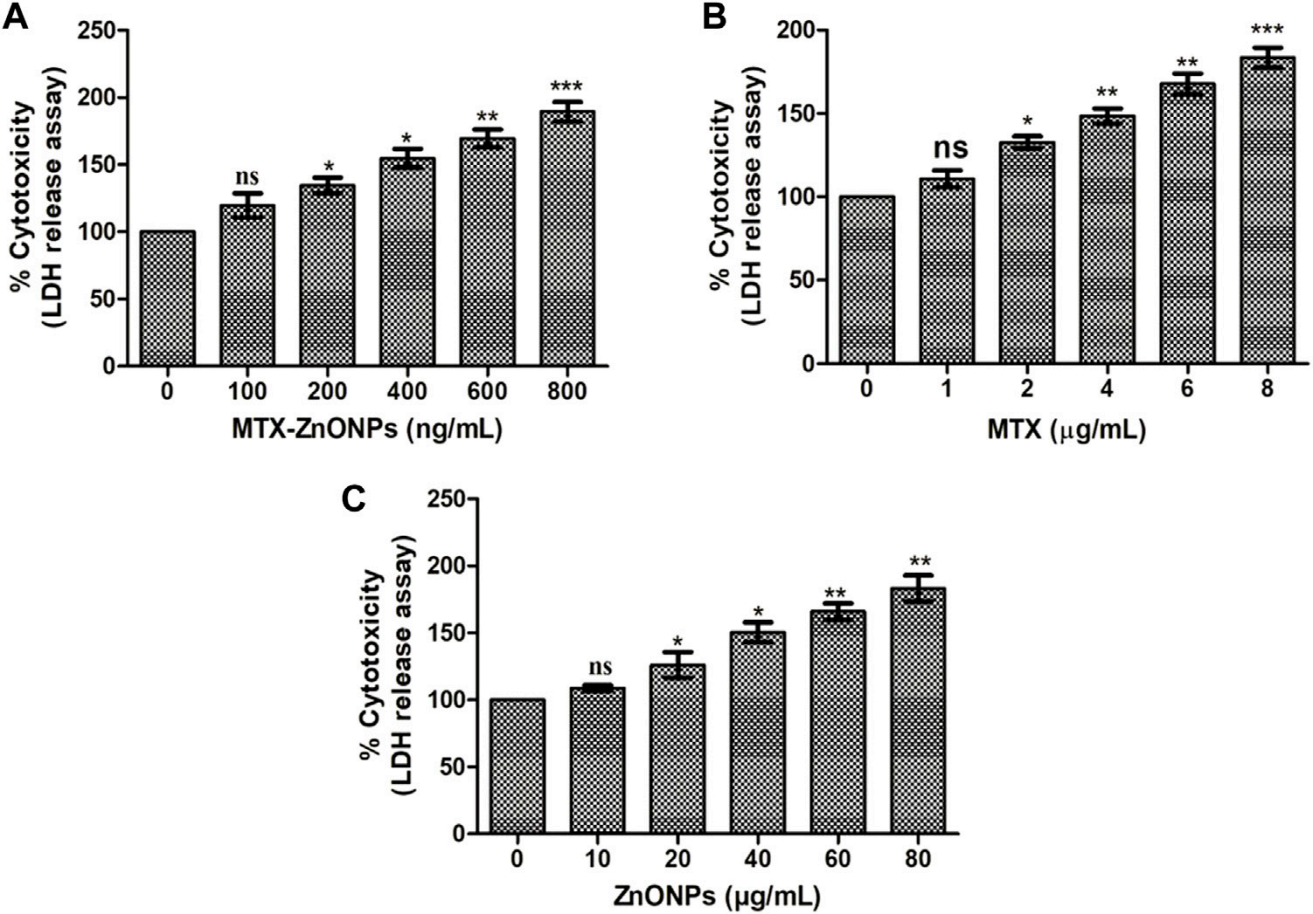
Percent cytotoxicity of A549 cells treated with different doses of **(A)** MTX-ZnONPs, **(B)** MTX alone, and **(C)** ZnONPs alone for 24 h assessed by LDH release assay. The results presented are the mean ± SEM of three independent experiments performed in triplicate. Significance among different dosage groups were determined using one-way ANOVA followed by Dunnett’s *post hoc* test (where *p < 0.05, **p < 0.01, and ***p < 0.001 represent significant differences compared with vehicle control).

The result of the LDH assay revealed that treatment of A549 cells with MTX-ZnONPs for 24 h resulted in a significant amount of cell death, and the percent cytotoxicity was found to be 19.52% ± 5.22%, 34.45% ± 3.37%, 54.78% ± 4.02%, 69.44% ± 3.84%, and 89.40% ± 4.14% at the doses of 100, 200, 400, 600, and 800 ng/mL, respectively (Figure 4A). Similarly, MTX alone also caused significant cell death in A549 cells, and the percent cytotoxicity was found to be 10.65% ± 2.96%, 32.58% ± 2.12%, 48.45% ± 2.60%, 67.77% ± 3.53%, and 83.40% ± 3.47% at the doses of 1, 2, 4, 6, and 8 μg/mL, respectively (Figure 4B). Furthermore, ZnONPs alone exerted a cytotoxic effect on A549 cells only at a dose of 10–80 μg/mL (Figure 4C). Thus, the aforementioned results further established that, at similar doses, drug-nanoconjugate treatment led to a greater amount of death in A549 cells compared to drug alone treatment. This assay also indicated the effective intracellular delivery of the drug via zinc oxide nanocarriers.

### Morphological alterations

**FIGURE 5:**
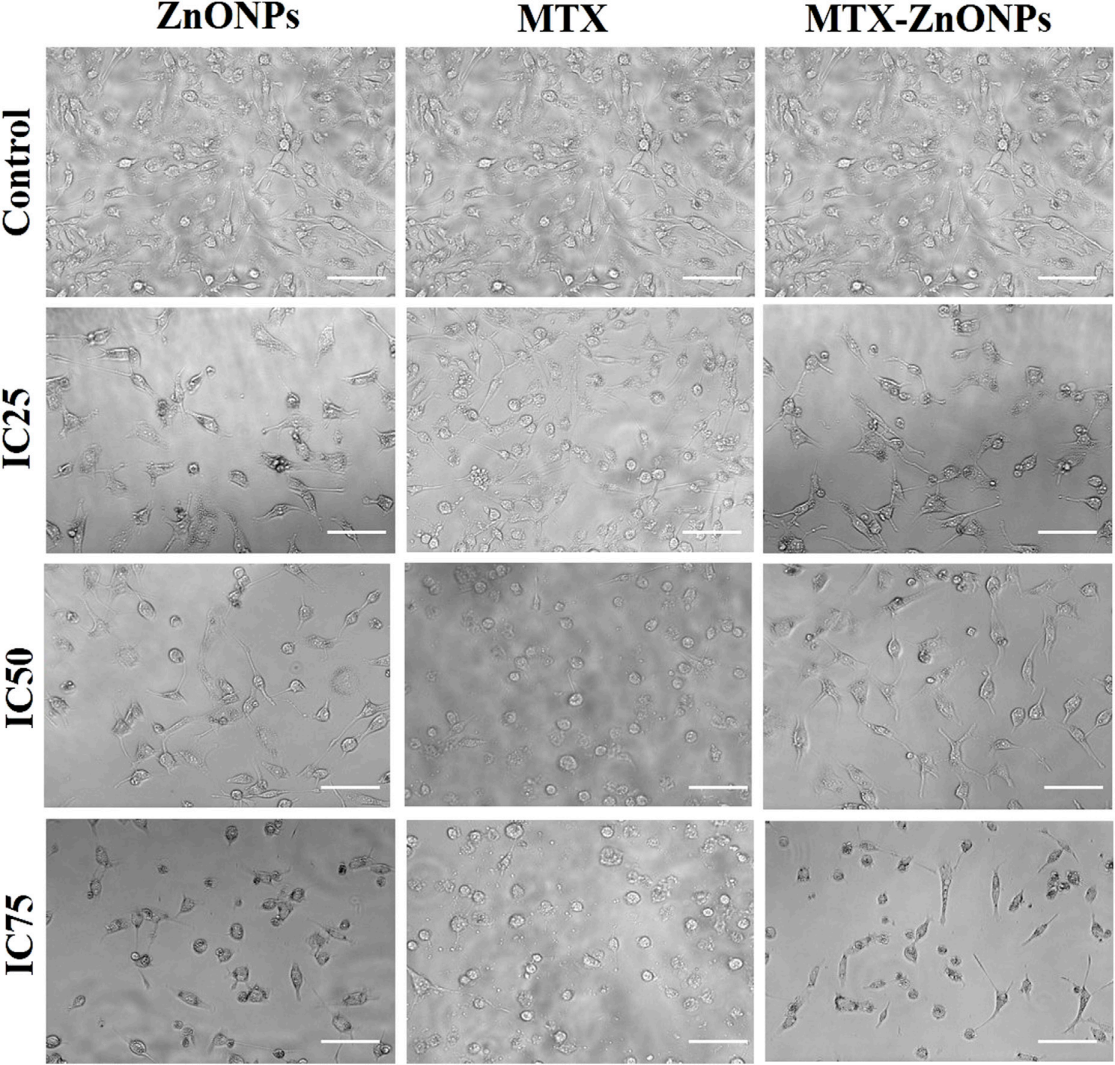
Cytomorphological images of A549 cells treated with IC_25_ (ZnONPs 27.83 μg/mL; MTX 1.95 μg/mL; and MTX-ZnONPs 182 ng/mL), IC_50_ (ZnONPs 65.30 μg/mL; MTX 3.58 μg/mL; and MTX-ZnONPs 327 ng/mL), and IC_75_ (ZnONPs 70.41 μg/mL; MTX 6.57 μg/mL; and MTX-ZnONPs 588 ng/mL) concentrations for 24 h analyzed by phase contrast microscopy. Images shown are representative of three independent experiments performed in triplicate (magnification ×20; scale bar 100 µm). The control image was reused in each treatment group.

To analyze morphological alterations in lung cancer cells, A549 cells were treated with isoeffective doses of MTX-ZnONPs, and MTX and ZnONPs alone. The morphology of A549 cells was significantly altered in all the treated groups after 24 h of treatment with IC_25_ (MTX-ZnONPs 182 ng/mL; MTX 1.95 μg/mL; and ZnONPs 27.83 μg/mL), IC_50_ (MTX-ZnONPs 327 ng/mL; MTX 3.58 μg/mL; and ZnONPs 65.3 μg/mL), and IC_75_ (MTX-ZnONPs 588 ng/mL; MTX 6.57 μg/mL; and ZnONPs 70.41 μg/mL) concentrations. A well-spread flattened morphology of cells was observed in the untreated control group, while a round morphology with cell shrinkage was observed in all the treatment groups. Membrane blebbing and lysis were also observed in some proportion of cells among all the treated groups, suggesting their cytotoxicity at the respective doses (Figure 5). As the treatment doses were increased from IC_25_ to IC_75_, the morphological alterations in A549 cells gradually increased in all the treated groups. As observed previously, MTX-ZnONPs induced similar changes in the cytomorphology of A549 cells at much lower doses compared with MTX, which also indicated the effective delivery of the drug by the carrier nanoparticles.

### Nuclear alterations

**FIGURE 6:**
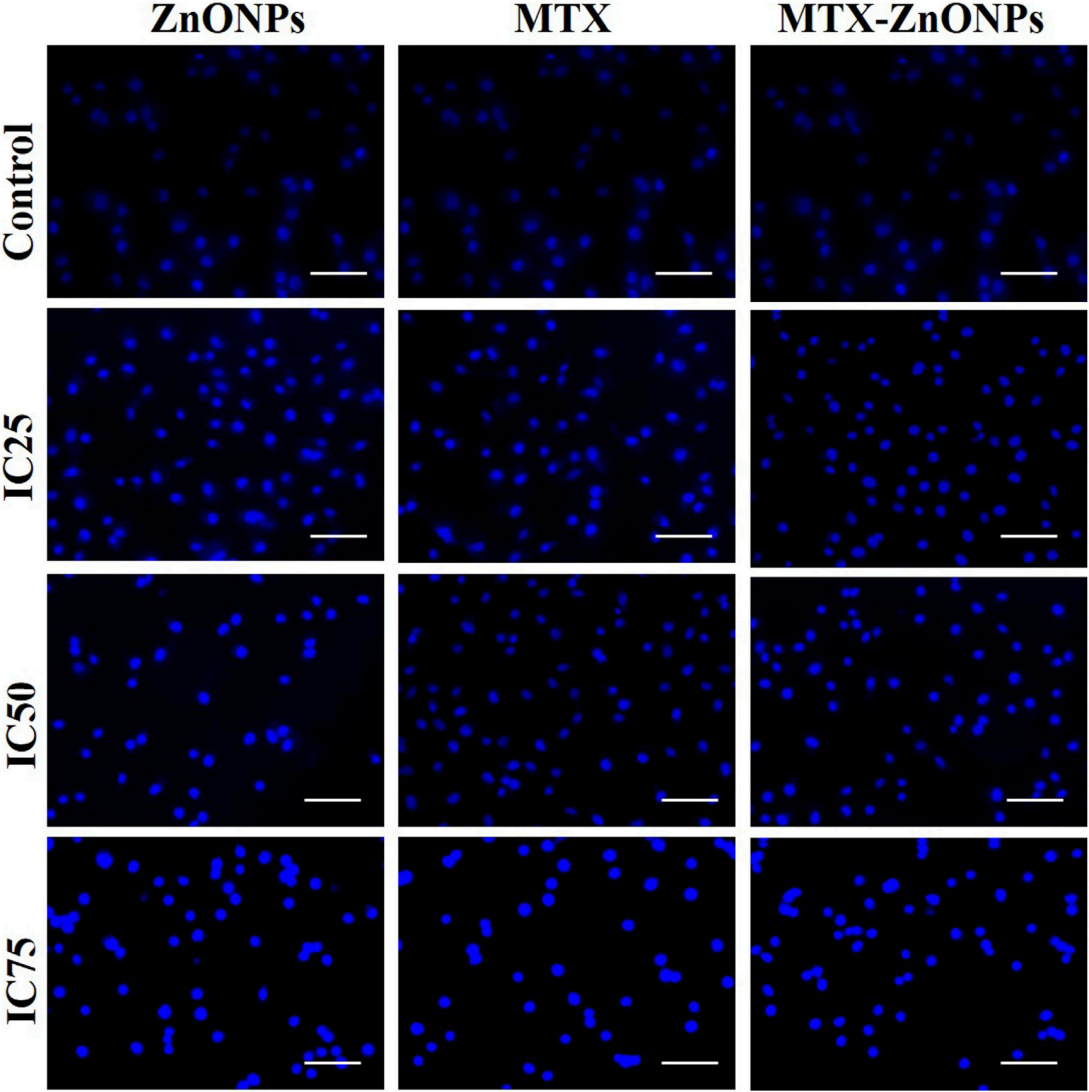
Nuclear morphology of DAPI-stained nuclei of A549 cells treated at IC_25_ (ZnONPs 27.83 μg/mL; MTX 1.95 μg/mL; and MTX-ZnONPs 182 ng/mL), IC_50_ (ZnONPs 65.30 μg/mL; MTX 3.58 μg/mL; and MTX-ZnONPs 327 ng/mL), and IC_75_ (ZnONPs 70.41 μg/mL; MTX 6.57 μg/mL; and MTX-ZnONPs 588 ng/mL) concentrations for 24 h analyzed by fluorescence microscopy. Images shown are representative of three independent experiments (scale bar: 100 μm; magnification: ×20). The control image was reused in each treatment group.

After treatment with IC_25_, IC_50_, and IC_75_ of MTX-ZnONPs, MTX alone, and ZnONPs alone for 24 h, substantial nuclear condensation and fragmentation in A549 cells were observed in all the treatment groups, whereas normal nuclear morphology was observed in the untreated control group (Figure 6). As the treatment doses were increased from IC_25_ to IC_75_, the nuclear alterations in A549 cells gradually increased in all the treatment groups. The result showed that MTX-ZnONPs exhibited corresponding nuclear changes in A549 cells at much lower concentrations compared to MTX. Furthermore, the nuclear condensation in A549 cells suggested the initiation of apoptosis induced by the drug.

### Disrupted mitochondrial membrane potential (ΔΨm)

**FIGURE 7:**
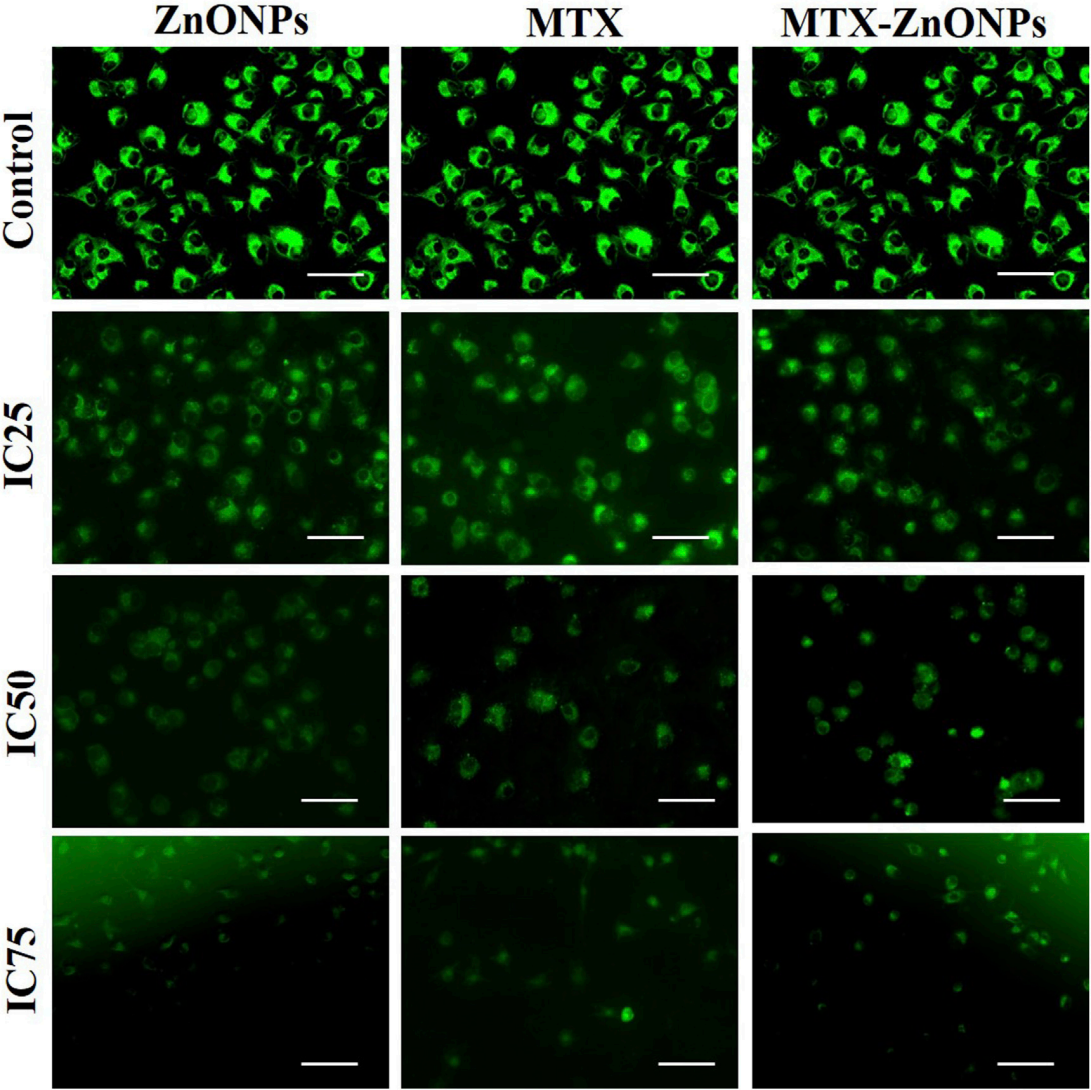
Qualitative assessment of mitochondrial membrane potential in Rhodamine 123-stained A549 cells treated at IC_25_ (ZnONPs 27.83 μg/mL; MTX 1.95 μg/mL; and MTX-ZnONPs 182 ng/mL), IC_50_ (ZnONPs 65.30 μg/mL; MTX 3.58 μg/mL; and MTX-ZnONPs 327 ng/mL), and IC_75_ (ZnONPs 70.41 μg/mL; MTX 6.57 μg/mL; and MTX-ZnONPs 588 ng/mL) concentrations for 24 h analyzed by fluorescence microscopy. Images shown are representative of three independent experiments (scale bar: 100 μm; magnification: ×20). The control image was reused in each treatment group.

After treatment with IC_25_, IC_50_, and IC_75_ of MTX-ZnONPs, MTX alone, and ZnONPs alone for 24 h, A549 cells exhibited a significant loss of ΔΨm in all the treatment groups (Figure 7). The fluorescence intensity of Rhodamine 123 in A549 cells, in all the treatment groups, was proportional to ΔΨm. Maximum fluorescence intensity was observed in the untreated control group, while reduced fluorescence was observed in all the treatment groups. As the treatment doses were increased from IC_25_ to IC_75_, the Rhodamine 123 fluorescence in A549 cells gradually decreased in all the treatment groups. Our result demonstrated that MTX-ZnONPs caused an equivalent reduction in ΔΨm in A549 cells at much reduced concentrations compared with MTX. Moreover, the loss of ΔΨm in A549 cells indicated the initiation of the intrinsic pathway of apoptosis caused by the aforementioned drug.

### Augmented ROS production

**FIGURE 8:**
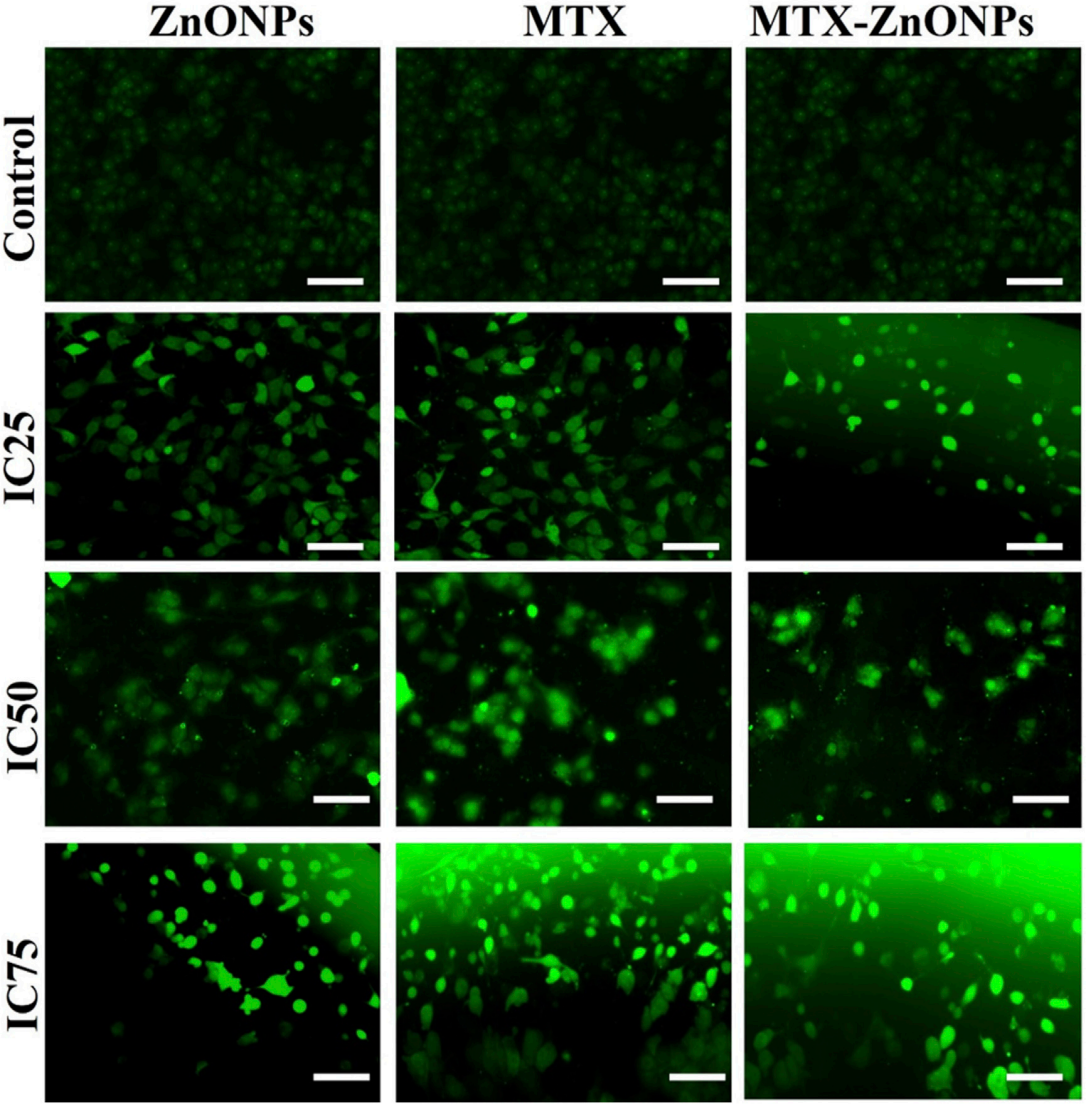
Qualitative evaluation of ROS in H_2_DCFDA-stained A549 cells treated at IC_25_ (ZnONPs 27.83 μg/mL; MTX 1.95 μg/mL; and MTX-ZnONPs 182 ng/mL), IC_50_ (ZnONPs 65.30 μg/mL; MTX 3.58 μg/mL; and MTX-ZnONPs 327 ng/mL), and IC_75_ (ZnONPs 70.41 μg/mL; MTX 6.57 μg/mL; and MTX-ZnONPs 588 ng/mL) concentrations for 24 h analyzed by fluorescence microscopy. Images shown are representative of three independent experiments (scale bar: 100 μm; magnification: ×20). The control image was reused in each treatment group.

The intracellular ROS level was found to be enhanced in A549 cells treated with IC_25_, IC_50_, and IC_75_ of MTX-ZnONPs, MTX alone, and ZnONPs alone for 24 h (Figure 8). The DCF fluorescence intensity in A549 cells was proportional to the level of ROS in all the treatment groups. The untreated control cells demonstrated insignificant fluorescence, while augmented fluorescence was observed in all the treatment groups. As the treatment doses were increased from IC_25_ to IC_75_, the intracellular ROS level in A549 cells gradually increased in all the treatment groups. The result showed that MTX-ZnONPs caused a similar increment in cellular ROS production in A549 cells at much lower concentrations compared to MTX. Moreover, the enhanced ROS production in A549 cells indicated the activation of the apoptotic pathway induced by the drug.

**FIGURE 9:**
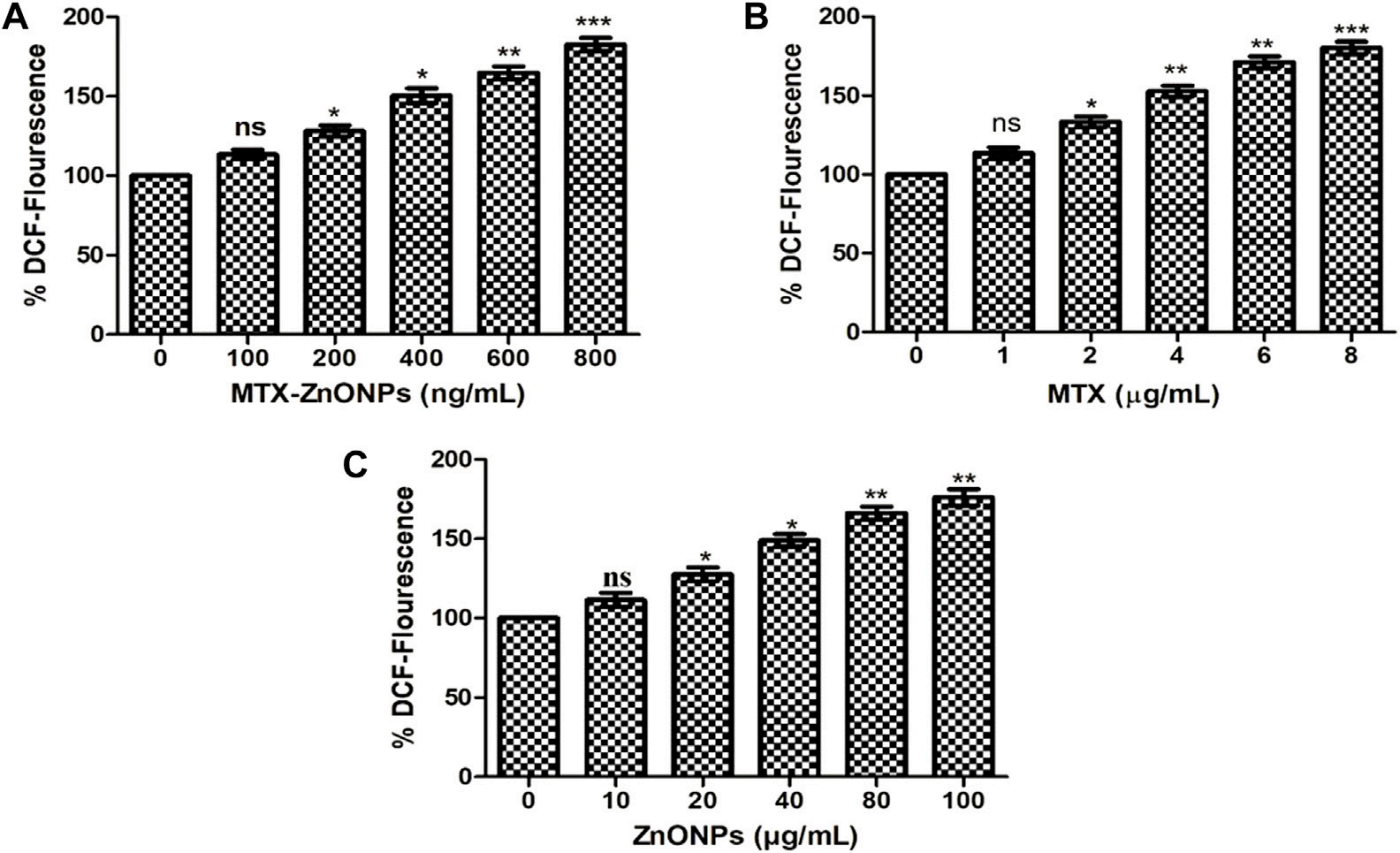
Quantitative determination of ROS in H_2_DCFDA-stained A549 cells treated with different concentrations of **(A)** MTX-ZnONPs, **(B)** MTX alone, and **(C)** ZnONPs alone for 24 h. The results presented are the mean ± SEM of three independent experiments performed in triplicate. Significance among different dosage groups were determined using one-way ANOVA followed by Dunnett’s *post hoc* test (where *p < 0.05, **p < 0.01, and ***p < 0.001 represent significant differences compared with vehicle control).

In an additional experiment, we also quantified the amount of ROS produced in lung cancer cells treated with MTX-ZnONPs and MTX and ZnONPs alone. The result demonstrated a dose-dependent increase of DCF fluorescence in A549 cells treated with MTX-ZnONPs, which was found to be 13.38% ± 2.96%, 28.29% ± 3.47%, 50.35% ± 4.68%, 64.84% ± 4.06%, and 82.62% ± 4.12% at the doses of 100, 200, 400, 600, and 800 ng/mL, respectively (Figure 9A). Similarly, MTX alone also caused a significant augmentation of DCF fluorescence in A549 cells, which was found to be 13.47% ± 3.49%, 33.34% ± 3.39%, 52.78% ± 3.49%, 71.21% ± 3.74%, and 80.17% ± 3.87% at the doses of 1, 2, 4, 6, and 8 μg/mL, respectively (Figure 9B). Furthermore, ZnONPs alone exhibited significant DCF fluorescence in A549 cells at a dose of 10–80 μg/mL (Figure 9C). Our result demonstrated that, at similar doses, MTX-ZnONP treatment led to the generation of a greater amount of ROS in A549 cells compared to treatment with MTX alone.

### NAC reduced cytotoxicity

**FIGURE 10:**
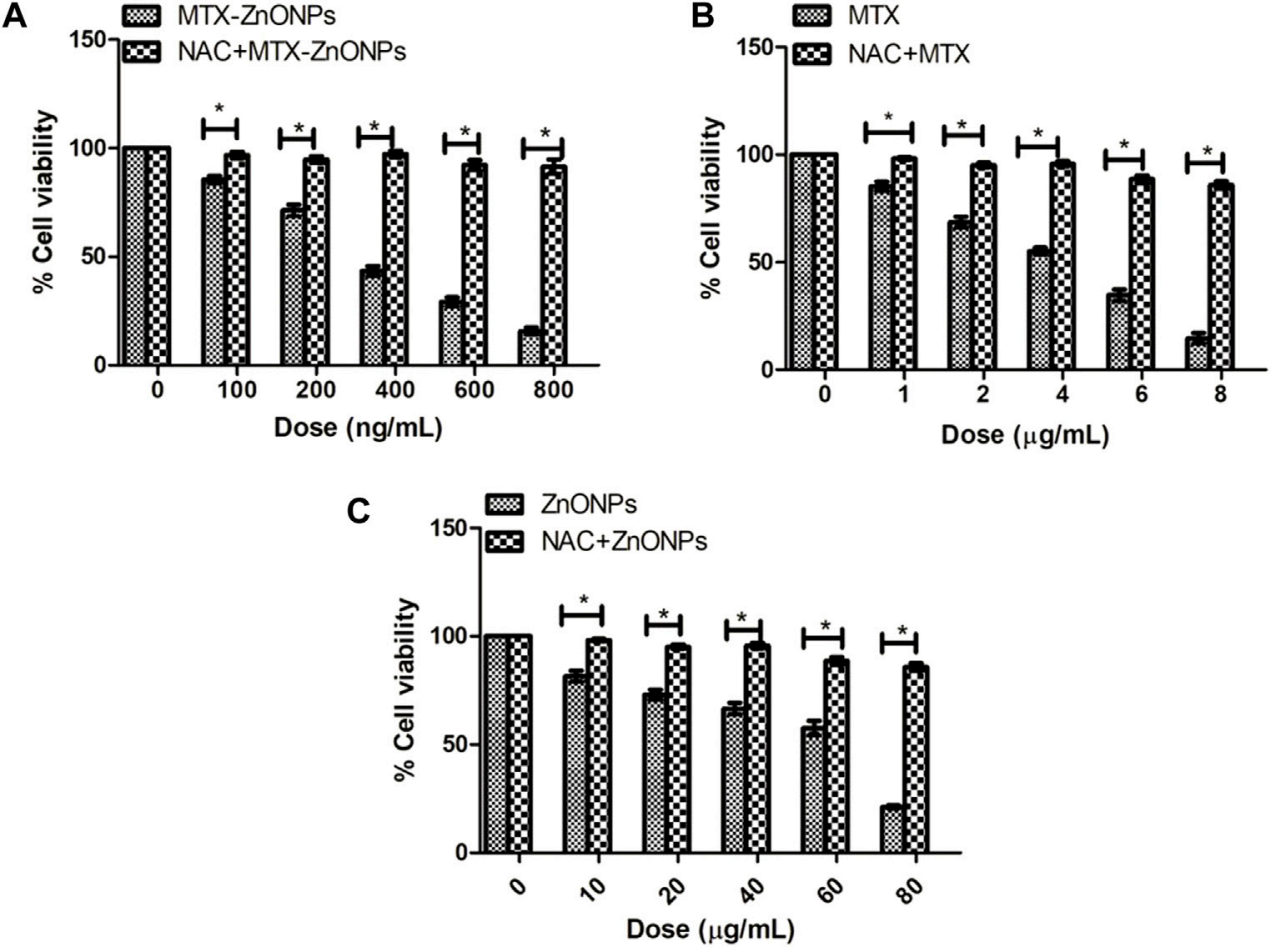
Percent cell viability of A549 cells, pretreated with NAC (5 mM), followed by treatment with different doses of **(A)** MTX-ZnONPs, **(B)** MTX alone, and **(C)** ZnONPs alone for 24 h assessed by MTT assay. The results presented are the mean ± SEM of three independent experiments performed in triplicate. Significance among two treatment groups were determined using two-tailed paired *t*-test (where *p < 0.05 represents significant difference between means of both treatment groups).

Pretreatment of NAC considerably reduced the cytotoxicity in A549 cells caused by MTX-ZnONPs, MTX alone, and ZnONPs alone (Figure 10). Our findings authenticated the fact that an enhanced level of ROS in all the treatment groups was critical for apoptosis in A549 cells. However, it was interesting to note that the cytotoxicity of MTX-ZnONPs, and MTX and ZnONPs alone, was not completely abrogated by NAC, which implicated the involvement of some ROS-independent apoptotic pathways in all the treatment groups.

### Activation of caspases

**FIGURE 11:**
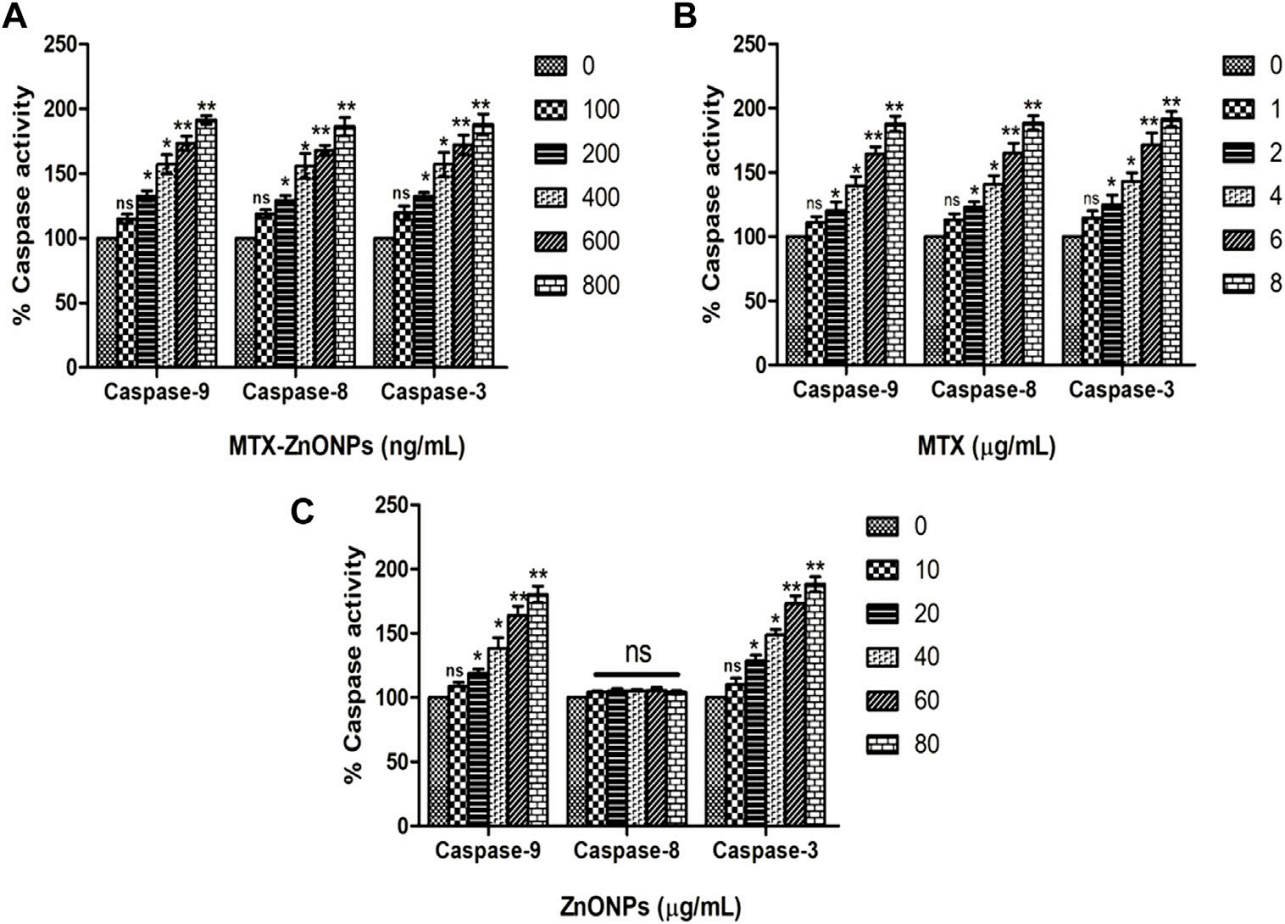
Percent caspase-9, -8, and -3 activities in A549 cells treated with different concentrations of **(A)** MTX-ZnONPs, **(B)** MTX alone, and **(C)** ZnONPs alone for 24 h. The results presented are the mean ± SEM of three independent experiments performed in triplicate. Significance among different dosage groups were determined using one-way ANOVA followed by Dunnett’s *post hoc* test (where *p < 0.05, **p < 0.01, and ***p < 0.001 represent significant differences compared with vehicle control).

To determine whether apoptosis in lung cancer cells was due to the activation of caspase-dependent or caspase-independent pathways, caspase activities were determined in A549 cells treated with the effective doses of MTX-ZnONPs and MTX and ZnONPs alone. The caspase-9 activity in the MTX-ZnONP-treated group was substantially augmented by 15.17% ± 2.01%, 32.70% ± 4.05%, 56.94% ± 4.33%, 73.50% ± 3.14%, and 91.54% ± 1.86% compared to the untreated control at the doses of 100, 200, 400, 600, and 800 ng/mL, respectively (Figure 11A). Similarly, MTX alone also exhibited significantly increased caspase-9 activity in A549 cells by 10.94% ± 2.81%, 20.03% ± 4.06%, 39.61% ± 4.14%, 64.17% ± 3.28%, and 87.87% ± 3.42% at the doses of 1, 2, 4, 6, and 8 μg/mL, respectively (Figure 11B). Furthermore, the activity of caspase-3 was significantly enhanced in MTX-ZnONP-treated A549 cells by 19.49% ± 3.13%, 32.41% ± 2.97%, 56.89% ± 5.43%, 72.35% ± 4.39%, and 88.08% ± 4.62% at the doses of 100, 200, 400, 600, and 800 ng/mL, respectively (Figure 11A). Similarly, MTX alone also exhibited substantially elevated caspase-3 activity in A549 cells by 14.49% ± 3.26%, 24.97% ± 4.23%, 42.81% ± 3.99%, 71.30% ± 5.37%, and 91.46% ± 3.44% at the doses of 1, 2, 4, 6, and 8 μg/mL, respectively (Figure 11B). The caspase-8 activity in A549 cells treated with MTX-ZnONPs was substantially augmented by 18.82% ± 1.80%, 29.30% ± 3.67%, 55.81% ± 5.64%, 67.96% ± 2.26%, and 86.33% ± 4.08% at the doses of 100, 200, 400, 600, and 800 ng/mL, respectively (Figure 11A). Likewise, MTX alone also exhibited significantly increased caspase-8 activity in A549 cells by 13.16% ± 2.68%, 22.97% ± 2.46%, 40.81% ± 3.96%, 65.23% ± 4.28%, and 88.66% ± 3.08% at the doses of 1, 2, 4, 6, and 8 μg/mL, respectively (Figure 11B). Additionally, ZnONPs alone did not exhibit any significant activity of caspase-8 in A549 cells after 24 h of treatment (Figure 11C). Our results demonstrated that, at similar doses, the MTX-ZnONP-treated group showed prominent caspase-9, -8, and -3 activities in A549 cells compared to that treated with MTX alone. Thus, our results established that MTX induced caspase-dependent apoptosis in lung cancer cells via the activation of both intrinsic and extrinsic pathways.

### Caspase inhibitors reduced the cytotoxicity

**FIGURE 12:**
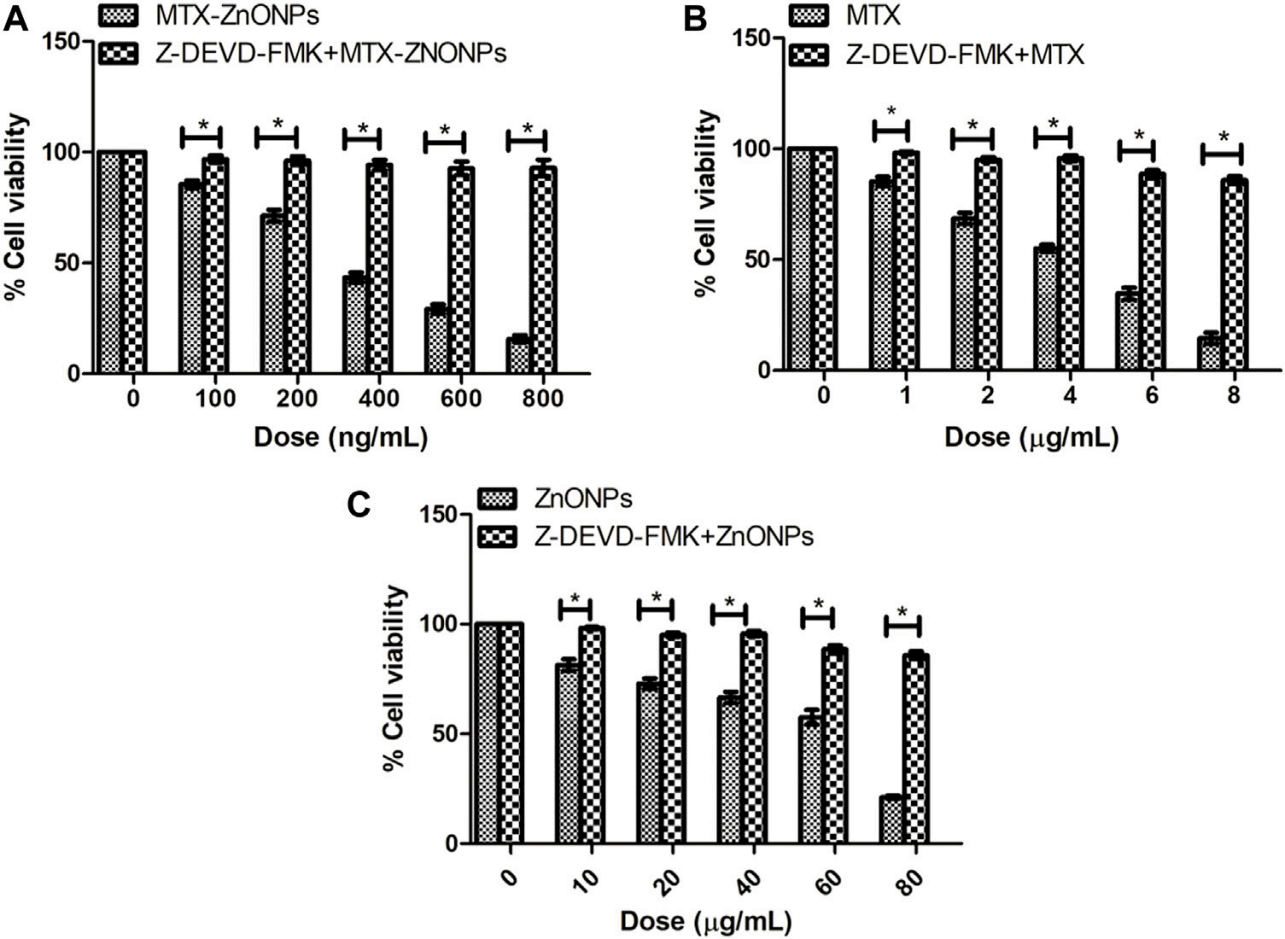
Percent cell viability of A549 cells, pretreated with caspase-3 inhibitor Z-DEVD-FMK (50 µM), followed by treatment with different doses of **(A)** MTX-ZnONPs, **(B)** MTX alone, and **(C)** ZnONPs alone for 24 h assessed by MTT assay. The results presented are the mean ± SEM of three independent experiments performed in triplicate. Significance among two treatment groups were determined using two-tailed paired *t*-test (where *p < 0.05 represents significant difference between means of both treatment groups).

Our results demonstrated that the caspase-3 inhibitor considerably attenuated the cytotoxicity in all the treatment groups, substantiating the activation of caspases in A549 cells (Figure 12). Interestingly, the caspase-3 inhibitor did not completely reduce the cytotoxicity in all the treatment groups, which indicates a minor role of a caspase-independent pathway of apoptosis in A549 cells along with the caspase-dependent pathway.

## Discussion

Since standard chemotherapeutic agents often exhibit serious side effects during chemotherapy, their dosage can be reduced by coupling them with some carrier or delivery agent to minimize the side effects of these drugs. This type of conjugation of drugs with some carrier will help deliver drugs directly to the cancer cells. Thus, a lower dose of the drug will be more effective than a higher therapeutic dose (Zhang et al., 2018). In this regard, nanotechnology can offer good drug-delivery options. The biocompatibility and exceptional physicochemical properties of ZnONPs have shown their potential in the diagnosis and treatment of various types of cancer (Hanley et al., 2008; Deng et al., 2009). ZnONPs have been shown to exhibit cytotoxicity in cancer cells by inducing intracellular ROS production and abrogation of ΔΨm, ultimately leading to the activation of caspases and apoptosis (Anjum et al., 2021). For the effective use of ZnONPs, their surface needs to be modified with certain capping (Kalpana et al., 2018; Xu et al., 2018). In this regard, the green synthesis of ZnONPs has received increasing attention recently (Lv et al., 2017). In the current study, biogenically synthesized ZnONPs were conjugated with the antifolate drug MTX, and their cytotoxic efficacy was determined against lung cancer cells.

We have discussed the biogenic synthesis of ZnONPs using a fungus, later identified as an *A. niger* strain, in the previous publication (Mishra et al., 2017; Mishra et al., 2022). In the current study, we have successfully shown the conjugation of MTX with biogenically synthesized ZnONPs. The conjugation of MTX with ZnONPs was authenticated by UV-vis spectroscopy, DLS, FTIR, and TEM. The drug-nanoconjugate showed high drug-loading efficiency, which indicated the efficiency of ZnONPs as drug carriers. We utilized EDC chemistry to conjugate MTX with ZnONPs. The drug–nanoparticle conjugates were highly stable due to the amide linkage of MTX with ZnONPs.

**FIGURE 13:**
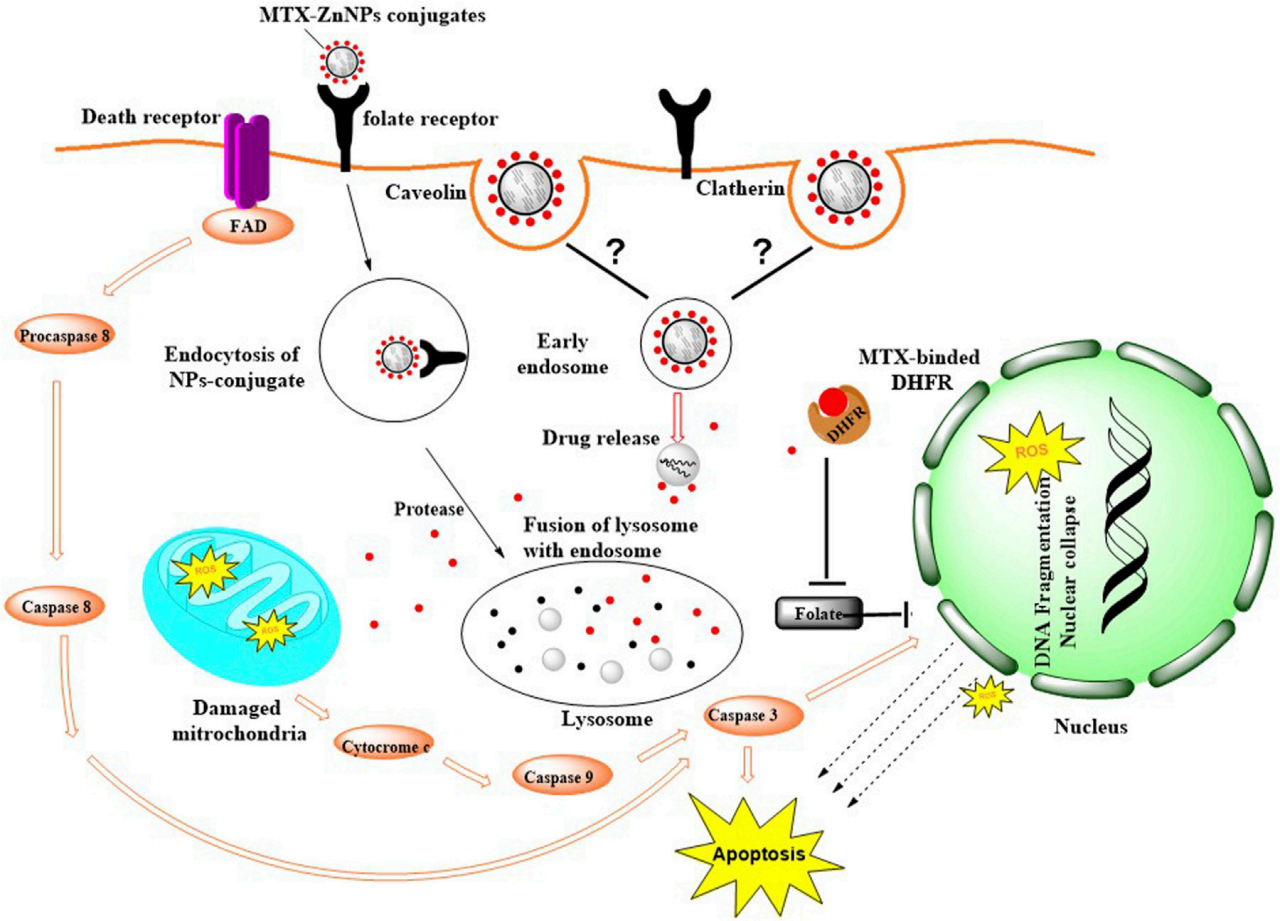
Possible mode of internalization of MTX-ZnONPs via caveolin-mediated or clathrin-mediated endocytosis and mechanism of action of MTX in A549 cells.

The cytotoxic efficacy of MTX-ZnONPs was further tested *in vitro* on a non-small cell lung cancer cell line, and the results of MTT and LDH release assays showed that MTX-ZnONPs (at the dose of 100–800 ng/mL) and MTX (at the dose of 1–8 μg/mL) were efficient in suppressing the growth of A549 cells; however, the efficacy of MTX-ZnONPs was found to be substantially enhanced at very low doses compared to that of MTX. The most plausible reason behind this enhanced activity of MTX-ZnONPs may be the high drug-loading efficiency of the biogenic nanocarrier and effective delivery of the drug, which could significantly increase the intracellular concentration of MTX, thus enhancing the inhibition of cancer cell proliferation. Interestingly, human folate receptors have been reported to take up MTX-loaded nanoparticles into cells to a better degree than MTX and are most likely to be released only in the acidic environment of lysosomes (Xie et al., 2018). Due to the high expression of the folate receptor on the target cancer cells, MTX-ZnONPs could possibly be internalized inside cancer cells easily and efficiently (Figure 13). Our results were in agreement with a previous study where MTX was conjugated with iron oxide nanoparticles (IONPs) as a drug delivery system to study its cytotoxic effect on cancer cells (Nosrati et al., 2018). Moreover, ZnONPs alone significantly inhibited the cell viability of A549 cells at a much higher concentration (at a dose of 10–80 μg/mL) compared to MTX-ZnONPs and MTX, which suggested the intrinsic cytotoxic properties of ZnONPs against lung cancer cells at higher concentrations. Previously, the cytotoxic property of ZnONPs was demonstrated in cancer cells, which corroborated our finding (Anjum et al., 2021). Furthermore, our results have also shown a significant cytotoxicity effect of ZnONPs against normal 3T3 cells at a dose range of 10–100 μg/mL (Supplementary Figure S1). This signified that ZnONPs are cytotoxic not only to A549 cells but also to 3T3 cells at this dose range (Pinho et al., 2020)**.**

When conjugated with MTX, these ZnONPs intriguingly exhibited a similar degree of effect at much lower doses (100–800 ng/mL). This observation might be due to drug alone or the nanocarrier. So, to confirm this observation, we again performed the cell viability assay of A549 cells treated with free ZnONPs at 100–800 ng/mL doses, which showed that ZnONPs were not cytotoxic to A549 cells at the indicated doses (Supplementary Figure S2). This result suggested the non-toxic behavior of free ZnONPs at much lower doses, which could be articulated to be its good nanocarrier property.

Furthermore, cytomorphological analysis performed at isoeffective concentrations by phase contrast microscopy revealed significant changes in the morphology of A549 cells in all the treatment groups, which were evident by cell shrinkage and detachment from the surface, forming clusters. Similarly, the nuclear changes in A549 cells in all the treatment groups, analyzed at isoeffective doses by a fluorescent dye DAPI, revealed fragmented and condensed nuclei, indicating initiation of apoptosis. Interestingly, at isoeffective concentrations, ZnONPs alone exerted similar effects on A549 cells compared to MTX-ZnONPs and MTX alone, which indicated the inherent cytotoxic properties of ZnONPs against lung cancer cells at higher concentrations (Figure 8). Moreover, the results also showed that MTX-ZnONPs exerted similar morphological changes in lung cancer cells at very low doses compared to MTX.

As described previously, the reduction in mitochondrial membrane potential (Δψ_m_) is an important event during the activation of the intrinsic pathway of apoptosis (Khan I. et al., 2018). Thus, Δψ_m_ in A549 cells in all the treatment groups, analyzed at isoeffective doses by using a cationic fluorescent dye Rhodamine 123, showed a gradual decrease. ZnONPs alone caused an equivalent reduction in Δψ_m_ in A549 cells compared to MTX-ZnONPs and MTX alone, which suggested the apoptotic potential of ZnONPs against lung cancer cells at higher doses. Furthermore, the results also showed that MTX-ZnONPs reduced the Δψ_m_ in lung cancer cells at very low concentrations compared to MTX, suggesting efficient delivery of the drug inside the cell.

Many anticancer drugs exert their therapeutic effect on cancer cells by augmenting the production of cytosolic ROS (Doroshow, 1986; Oh et al., 2007). ROS have been implicated in many cellular functions, such as apoptosis (Nakazato et al., 2007). Results of qualitative analysis of ROS, analyzed at isoeffective doses in A549 cells in all the treatment groups, depicted an augmented intracellular ROS level. Intriguingly, ZnONPs alone induced a similar amount of ROS generation in A549 cells compared to MTX-ZnONPs and MTX at isoeffective doses, which suggested that ZnONPs alone caused ROS-dependent apoptosis in lung cancer cells at higher doses. Furthermore, the results also showed that MTX-ZnONPs induced a similar amount of ROS in lung cancer cells at very low concentrations compared to MTX. Thus, our results suggested that MTX primarily caused ROS-mediated cell death in A549 cells.

Additionally, the results of quantitative analysis of ROS revealed that MTX-ZnONPs (at the dose of 100–800 ng/mL) and MTX alone (at the dose of 1–8 μg/mL) were effective in augmenting the intracellular ROS level in A549 cells; however, the effect of MTX-ZnONPs was more profound at very low doses compared to that of MTX. Moreover, ZnONPs alone substantially elevated the ROS level in A549 cells at much higher concentrations (10–80 μg/mL) compared to MTX-ZnONPs and MTX alone, which illustrated that the cytotoxic property of ZnONPs was due to ROS-mediated programmed cell death in lung cancer cells at higher concentrations. To corroborate the production of ROS in all the treatment groups, cell survival was estimated in lung cancer cells pretreated with NAC. It was observed that NAC significantly ameliorated the cell viability in all the treatment groups, authenticating the crucial role of ROS-mediated apoptosis in A549 cells. Interestingly, NAC did not completely ameliorate the survival of A549 cells in all the treatment groups, which indicated the role of ROS-independent pathways of apoptosis in A549 cells. Thus, our results showed that MTX could induce apoptosis in lung cancer cells via both ROS-dependent and ROS-independent pathways.

It has been well established that caspase-8 and caspase-9 play indispensable roles in the activation of the extrinsic and intrinsic apoptotic pathways, respectively (Ona et al., 1999). Moreover, caspase-3 is the chief executor of apoptosis by cleaving and dismantling the cellular structures (Bakar et al., 2010). Therefore, to confirm whether the cell death induced in lung cancer cells in all the treatment groups was due to the extrinsic or intrinsic pathway of apoptosis, caspase activities were determined in A549 cells. The results showed that MTX-ZnONPs (at the dose of 100–800 ng/mL) and MTX alone (at the dose of 1–8 μg/mL) caused significant activation of caspase-9, -8, and -3 in A549 cells; however, the effect of MTX-ZnONPs was more profound at very low doses compared to that of MTX. These results suggested that MTX induced apoptosis in A549 cells via both extrinsic and intrinsic pathways. Moreover, ZnONPs alone substantially activated caspase-9 and -3 in A549 cells at much higher concentrations (10–80 μg/mL) compared to MTX-ZnONPs (at the dose of 100–800 ng/mL) and MTX alone (at the dose of 1–8 μg/mL), while there was no significant activation of caspase-8, illustrating the activation of only the mitochondrial pathway of programmed cell death in lung cancer cells treated with ZnONPs at higher concentrations.

To validate the activation of caspases in lung cancer cells, cell survival was estimated in all the treatment groups pretreated with the caspase-3 inhibitor. The results showed that pre-treatment with the caspase-3 inhibitor significantly ameliorated the cell viability, demonstrating the instigation of caspases during programmed cell death in A549 cells in all the treatment groups. Intriguingly, pretreatment with a caspase-3 inhibitor did not completely ameliorate cell viability in lung cancer cells, indicating the activation of a caspase-independent pathway of apoptosis in A549 cells. Thus, our results showed that MTX could induce apoptosis in lung cancer cells via both caspase-dependent and caspase-independent pathways.

## Conclusion

In conclusion, the results of the current study have illustrated the conjugation of MTX with ZnONPs with high drug-loading efficiency. Moreover, the cytotoxic efficiency of MTX-ZnONPs was found to be enhanced compared to MTX alone in lung cancer cells. ZnONPs exhibited significant toxicity against lung cancer cells only at very high doses compared to the effective doses of drug-nanoconjugates, which suggested that the high therapeutic efficiency of MTX-ZnONPs was due to intracellular delivery of MTX. Thus, our results emphasized the efficient delivery of MTX to lung cancer cells via ZnONPs as nanocarriers.

## Data Availability

The original contributions presented in the study are included in the article/Supplementary Material; further inquiries can be directed to the corresponding authors.

## Supplementary material

SUPPLEMENTARY FIGURE S1 Percent cell viability of 3T3 cells treated with different doses of ZnONPs for 24 h assessed by MTT assay. The results presented are the mean ± SEM of three independent experiments performed in triplicate. Significance among different dosage groups were determined using one-way ANOVA followed by Dunnett’s *post hoc* test (where **p* <0.05, ***p* <0.01, and ****p* <0.001 represent significant differences compared with vehicle control).

SUPPLEMENTARY FIGURE S2 Percent cell viability of A549 cells in all the treatment groups treated with the effective doses of MTX-ZnONPs (100–800 ng/mL) for 24 h. The results presented are the mean ± SEM of three independent experiments performed in triplicate. Significance in different treatment groups at different doses were determined using two-way ANOVA with Bonferroni’s *post hoc* tests (where **p* <0.05, ***p* <0.01, and ****p* <0.001 represent significant differences between means of different treatment groups at different doses).
