# Corrigendum: Methotrexate-conjugated zinc oxide nanoparticles exert substantially improved cytotoxic effect on lung cancer cells by inducing apoptosis

**DOI:** 10.3389/fphar.2024.1423402

**Published:** 2024-07-29

**Authors:** Prakriti Mishra, Mohammad Faizan Ali Ahmad, Lamya Ahmed Al-Keridis, Mohd Saeed, Nawaf Alshammari, Nadiyah M. Alabdallah, Rohit Kumar Tiwari, Afza Ahmad, Mahima Verma, Shireen Fatima, Irfan Ahmad Ansari

**Affiliations:** ^1^ Department of Biosciences Integral University Lucknow, Lucknow, India; ^2^ Biology Department, Faculty of Science, Princess Nourah Bint Abdulrahman University, Riyadh, Saudi Arabia; ^3^ Department of Biology, College of Science, University of Hail, Hail, Saudi Arabia; ^4^ Department of Biology, College of Science, Imam Abdulrahman Bin Faisal University, Dammam, Saudi Arabia; ^5^ Basic and Applied Scientific Research Centre, Imam Abdulrahman Bin Faisal University, Dammam, Saudi Arabia; ^6^ Department of Clinical Research, School of Allied Health Sciences, Sharda University, Uttar Pradesh, India

**Keywords:** zinc oxide, nanoparticles, methotrexate, lung cancer, A549, reactive oxygen species, caspases

In the published article, there was an error in Figure 8 as published. Partial overlap between the IC50-ZnONPs, IC50-MTX, and IC50-MTX-ZnONPs panels were unintentionally duplicated. The corrected Figure 8 and its caption appear below.

**FIGURE 8:**
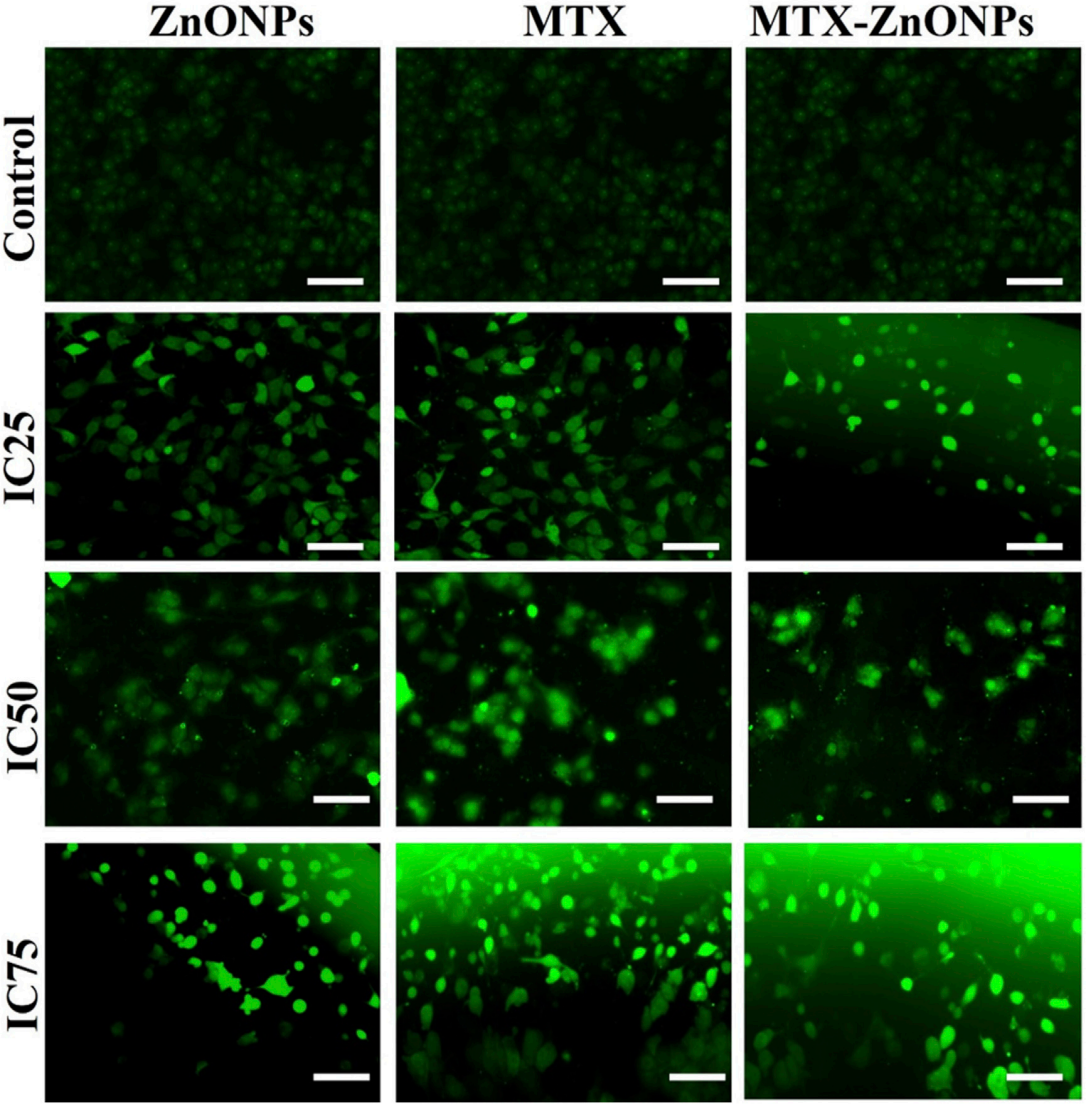
Qualitative evaluation of ROS in H2DCFDA-stained A549 cells treated at IC25 (ZnONPs 27.83 μg/mL; MTX 1.95 μg/mL; and MTX-ZnONPs 182 ng/mL), IC50 (ZnONPs 65.30 μg/mL; MTX 3.58 μg/mL; and MTX-ZnONPs 327 ng/mL), and IC75 (ZnONPs 70.41 μg/mL; MTX 6.57 μg/mL; and MTX-ZnONPs 588 ng/mL) concentrations for 24 h analyzed by fluorescence microscopy. Images shown are representative of three independent experiments (scale bar: 100 μm; magnification: ×20). The control image was reused in each treatment group.

The authors apologize for this error and state that this does not change the scientific conclusions of the article in any way. The original article has been updated.

