# Supplementary figures and images for: Methotrexate-conjugated zinc oxide nanoparticles exert a substantially improved cytotoxic effect on lung cancer cells by inducing apoptosis
Source: Front Pharmacol. 2023 Oct 17;14:1194578. doi: 10.3389/fphar.2023.1194578 (PMC10616591; doi:10.3389/fphar.2023.1194578)

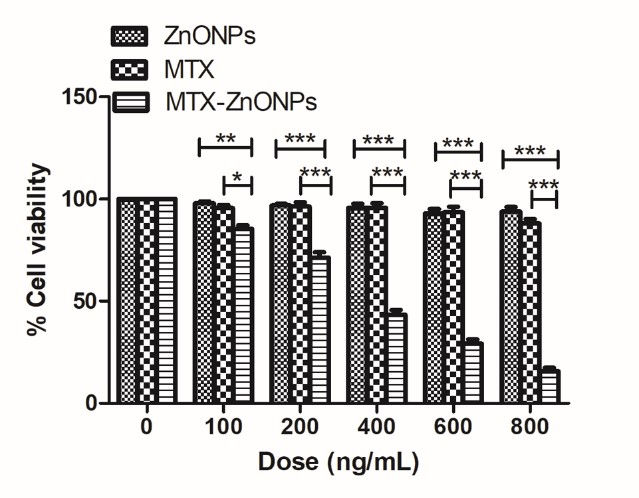

Supplement: Supplementary file 1 [file Image2.JPEG]

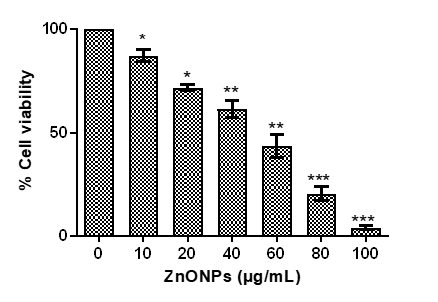

Supplement: Supplementary file 2 [file Image1.TIF]
